# Multicenter evaluation of the Acaruiter Respiratory Panel for diagnosis of respiratory tract infections in Chinese children

**DOI:** 10.1128/spectrum.00589-23

**Published:** 2023-10-09

**Authors:** Linqing Zhao, Xiaoyan Dong, Ling Cao, Yongmei Jiang, Hong Zhang, Ran Mo, Hanmin Liu

**Affiliations:** 1 Laboratory of Virology, Beijing Key Laboratory of Etiology of Viral Diseases in Children, Capital Institute of Pediatrics, Yabao Road, Chaoyang District, Beijing, China; 2 Shanghai Children's Hospital Affiliated to Shanghai Jiaotong University, Shanghai, China; 3 Department of Pediatrics, West China Second University Hospital, Sichuan University, Chengdu, China; 4 Nanjing Drum Tower Hospital, The Affiliated Hospital of Nanjing University Medical School, Nanjing, China; ASCR, Ceske Budejovice, Czech Republic

**Keywords:** children, respiratory tract infection, multiplex PCR assay, pathogen

## Abstract

Respiratory tract infection represents the most frequent infectious disease afflicting children worldwide. This study was to evaluate the performance of Acaruiter Respiratory Panel (fluorescent PCR) in the detection of respiratory tract infection pathogens in children. Clinical trials were conducted in three representative pediatric hospitals in Shanghai, Chengdu, and Beijing. With reference to another widely used multiplex assay and first-generation sequencing, the sensitivity, specificity, accuracy, and consistency coefficient kappa (*κ*) value of the assay were evaluated to verify the accuracy and effectiveness in the detection of clinical respiratory pathogens, so as to objectively evaluate the clinical performance and application value of Acaruiter Respiratory Panel (fluorescent PCR). A total of 1,540 samples were screened in this clinical trial project, of which 1,404 were valid samples. Based on the comprehensive analysis of the clinical trial data of 1,404 effective samples, the detection ability of Acaruiter Respiratory Panel (fluorescent PCR) for various pathogens compared with the results of clinical diagnosis, the positive percent agreement was 97.04%–100%, the negative percent agreement was 99.31%–100%, the total percent agreement was 99.36%–100%, and the kappa value was 0.982–1.000, which had good consistency and high accuracy.

**IMPORTANCE:**

Compared to multiplex PCR assays that are available in the Chinese market, the Acaruiter Respiratory Panel (fluorescent PCR) covers a wider range of pathogens including eight viruses, five bacteria, *Mycoplasma pneumoniae* and *Chlamydia pneumoniae*, and has high accuracy and effectiveness in the detection of pathogens. This is the first study to evaluate the performance of the Acaruiter Respiratory Panel. This regent may be a promising assay for comprehensive testing for respiratory pathogens from nasopharyngeal swab specimens in Chinese children.

## INTRODUCTION

Respiratory tract infection in children is one of the most common diseases worldwide and the most common infectious disease in children (1). Infected children can have different symptoms such as rhinitis, runny nose, nasal congestion, cough, mild pharyngitis, and generalized fever, which can lead to serious comorbidities and even death. Acute respiratory infection is the most common cause of illness and death in children under 5 years of age. In China, the fatality rate of pneumonia in children under 5 years old is 167.2/100,000, which ranks first in the number of deaths from the disease, while the proportion of viruses in the pathogens of severe pneumonia in children under 5 years old is 61.4% (2). Children’s respiratory tract infection is divided into upper respiratory tract infection and lower respiratory tract infection. The pathogens causing children’s respiratory tract infections are complex and diverse, and the etiology of infection is complex, mainly including bacteria (3), viruses (4), and some atypical pathogens, such as *Mycoplasma pneumoniae* and *Chlamydia pneumoniae*. Different pathogens can show similar symptoms and imaging changes, but the symptoms and imaging changes of the same pathogen infection are heterogeneous, so the diagnosis of respiratory tract infection is very difficult. It is difficult to distinguish the causes of infection according to symptoms and imaging images (5) and treat pathogens in time. Due to the particularity of children, if it is not diagnosed in time at the initial stage of the disease, it will often lead to some long-term sequelae. Therefore, rapid and accurate detection of pathogens of respiratory tract infection is the basis for timely control of infection (6). At the same time, it can reduce the hospitalization time and unnecessary use of antibiotics in children (7). During the epidemic of COVID-19, the rapid and accurate detection of pathogens of respiratory tract infection is beneficial to the timely triage of children with respiratory infectious diseases in outpatient and emergency departments, provides a reasonable and accurate basis for diagnosis and treatment, and plays an irreplaceable role (8).

The first multiplex respiratory panel was cleared by the Food and Drug Administration (FDA) in 2009. This has been followed by a number of such syndromic assays. These panels vary in the number of analytes detected and the time to result, but most are designed to be simple to use and require little hands-on time (9). At present, among the kits that have been listed in China, the coverage of common pathogens is relatively low. Health Respiratory Panel covers the widest range of pathogens, but in clinical application, it is still unable to cover common bacterial infections, which will affect the overall clinical evaluation of patients' infections. Most respiratory infections in children are multiple, mainly the compound infection of bacteria and viruses, and the combination of pathogens is often complex and irregular (10, 11). If the virus, bacteria, *Mycoplasma pneumoniae*, or *Chlamydia pneumoniae* are detected alone, it is impossible to accurately evaluate the infection of the patient, thus affecting the final diagnosis and treatment of the patient.

Acaruiter Respiratory Panel is a new rapid highly multiplexed PCR-based assay for the diagnosis of respiratory tract infections. In this study, Acaruiter Respiratory Panel was used as the assessment reagent, and Health Respiratory Panel and the first-generation sequencing were used as the comparator testing to verify the clinical application performance of the Acaruiter Respiratory Panel.

## MATERIALS AND METHODS

### Study design and patients

**Fig 1:**
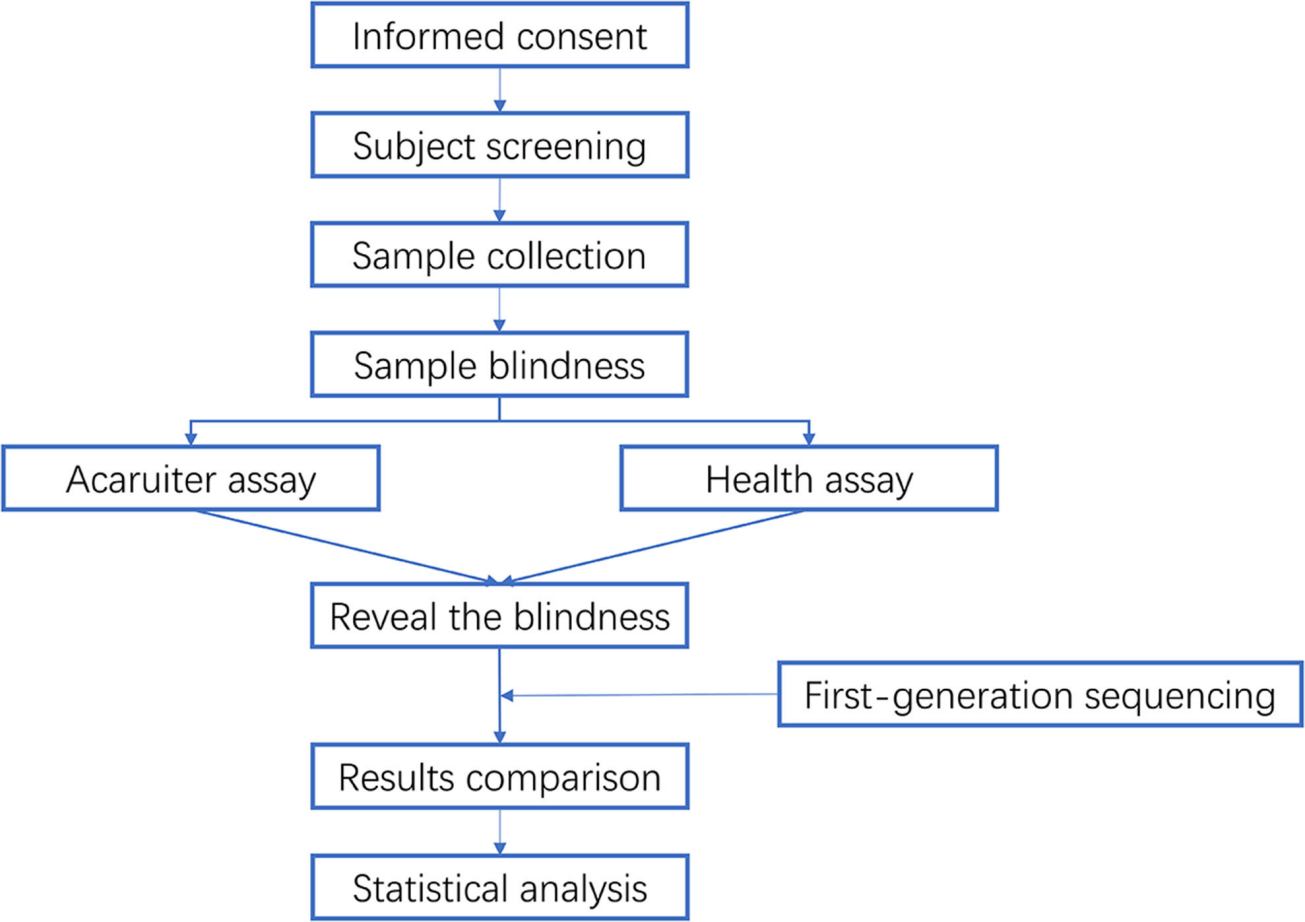
The research design of blindness and workflow.

This clinical trial adopted a multicenter, blind, and paired trial design. Pharyngeal swab samples were obtained from outpatients and inpatients of Children’s Hospital of Shanghai (Center 01, Ethical approval: 2020I157), West China Second Hospital, Sichuan University (Center 02, Ethical approval: Q2020008), and Children’s Hospital affiliated with Capital Institute of Pediatrics [Center 03, Ethical approval: (2021-003)] from March to November 2021. The specimens were tested with Acaruiter Respiratory Panel (fluorescent PCR method) product. Taking Health Respiratory Panel and first-generation sequencing method as the standard, the accuracy and effectiveness of the Acaruiter Respiratory Panel in clinical detection were verified, and the clinical application performance of the reagent was evaluated. The research design of blindness and workflow is shown in Fig. 1.

### Inclusion criteria

1. Subjects with symptoms of respiratory infection, including but not limited to cough, nasal congestion, nasal leakage, sore throat, fever, headache, and myalgia.
2. Subjects with similar symptoms as above or a history of close contact with respiratory diseases.
3. Residual samples of pharyngeal swabs left from previous respiratory tract treatment in subjects with a residual sample volume (preservation fluid) of at least 800 µL.

### Exclusion criteria

1. Samples not collected and preserved as required.
2. Samples with insufficient volume for nucleic acid extraction.
3. Samples not recorded with the required sample information.
4. Samples with missing patient diagnostic information or other factors that make sample information untraceable.

### Sample collection and preservation

#### Sample collection

Pharyngeal swab samples that met the collection requirements in clinical trial institutions were collected in accordance with the instructions, and the basic information about the samples was recorded. The sample collection method was as follows. Medical staff pressed the patient’s tongue with the tongue depressor with the left hand, extended the swab to the pharyngeal isthmus with the right hand, wiped the posterior pharyngeal wall and both tonsils with moderate force several times, and rotated the swab to increase the contact surface, avoiding contact with the tongue and oral mucosa. The swab was put into the sampling tube with 3 mL of collection solution quickly after sampling.

#### Sample preservation

Specimen was collected and sent to the testing laboratory immediately. Samples that could not be tested immediately should be stored under appropriate conditions. The swab of the secretion to be tested was stored at 2–8°C and the storage period should not exceed 72 h; the storage period was half a year at −20°C ± 5°C and the long-term storage temperature was below −70°C. The frozen sample should be restored to room temperature before testing and the sample should avoid repeated freezing and thawing.

### Acaruiter Respiratory Panel

**Fig 2:**
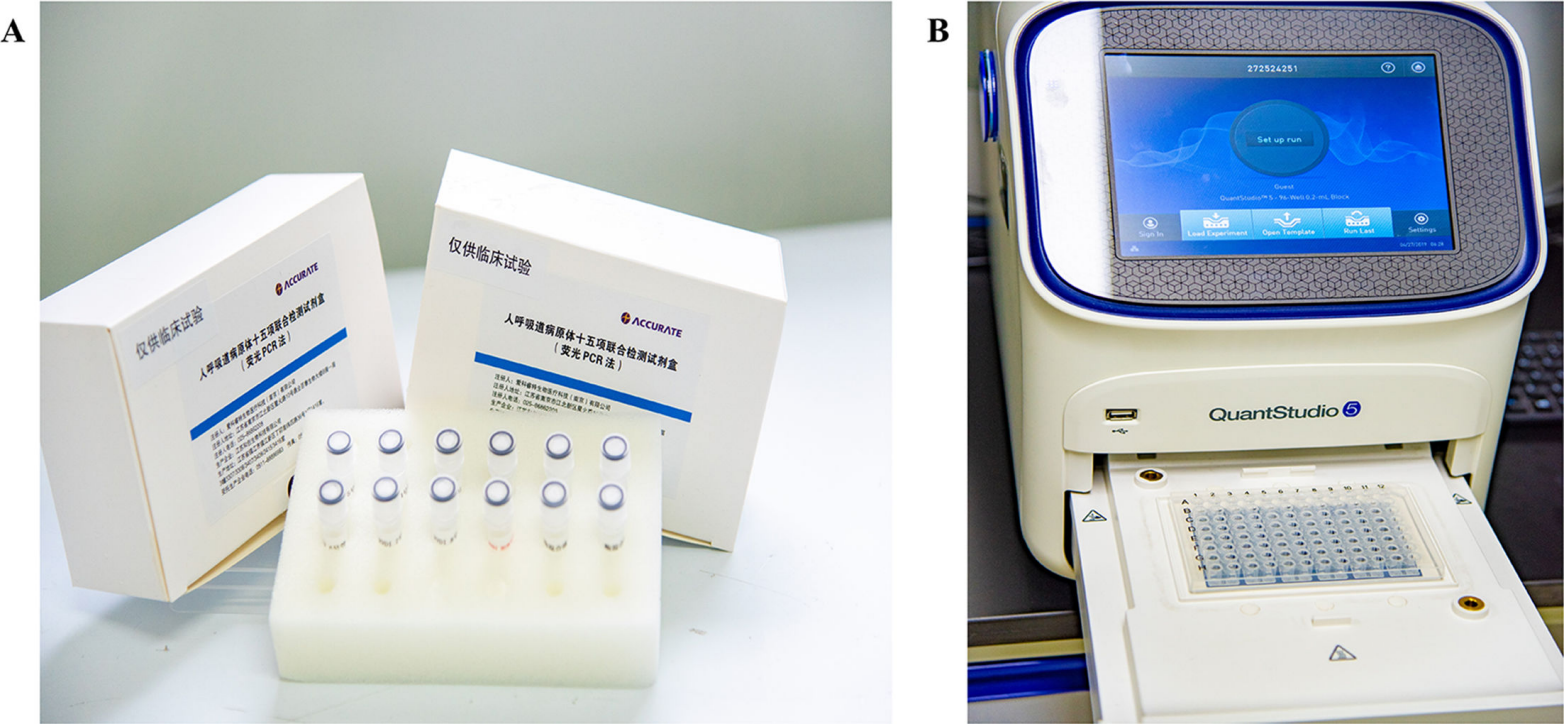
Acaruiter Respiratory Panel (A) and PCR instrument (B).

The nucleic acid test was performed with Acaruiter Respiratory Panel (Fig. 2A). The assay uses real-time fluorescent quantitative PCR-TaqMan fluorescent probe technology. When the probe is intact, the fluorescent signal emitted by the reporter group is absorbed by the quenched group. During PCR amplification, Taqase hydrolyzes the probe, separating the signal group from the quenched group, and the signal group fluoresces. For each DNA strand amplified, a fluorescent molecule is formed. PCR products are formed while the fluorescence signal is accumulated, and the detection of the target pathogens in the sample can be achieved by reading the intensity of the fluorescence signal by the instrument (Fig. 2B). The assay uses multi-channel fluorescence detection mode for qualitative analysis of nucleic acids of 15 human respiratory pathogens as an aid in the diagnosis of clinical human respiratory infections. The following pathogen types and subtypes are identified: influenza A virus, influenza B virus (strains V and Y), human adenovirus (1, 2, 3, 4, 5, 7), human rhinovirus (A, B, C), human parainfluenza virus I, II, III, human coronavirus (229E, OC43, NL63, HKU1), respiratory syncytial virus (A, B), human metapneumovirus (A, B), *Mycoplasma pneumoniae*, *Chlamydia pneumoniae*, *Haemophilus influenzae*, *Group A Streptococcus*, *Bordetella pertussis*, *Staphylococcus aureus*, and *Streptococcus pneumoniae*.

### Comparator testing

Comparator testing consisted of Health Respiratory Panel and first-generation sequencing method. Health Respiratory Panel can detect pathogens including influenza A virus (H7N9, H1N1, H3N2, H5N2), influenza A virus H1N1 (2009), seasonal H3N2 virus, influenza B virus (V strain and Y strain), human adenovirus (group B, groups C and E), Boca virus, human rhinovirus, human parainfluenza virus (types 1, 2, 3, and 4), human coronavirus (229E, OC43, NL63, and HKU1), respiratory syncytial virus (A and B), human metapneumovirus, *Mycoplasma pneumoniae*, and *Chlamydia trachomatis*. The detection results of human adenovirus, human parainfluenza virus, human coronavirus, respiratory syncytial virus, and *Chlamydia pneumoniae* were not classified. In this study, the first-generation sequencing was utilized as the reference standard, and a comparative analysis was performed to compare the findings from the two panels against those derived from first-generation sequencing. First-generation sequencing was performed by Sangon Biotech (Shanghai) Company Limited on a 3730 xl DNA Analyzer using the BigDye Terminator v3.1 kit (Thermo Fisher).

Besides, viral or bacterial culture methods were also applied where conditions permitted. Similarly, the results of Acaruiter Respiratory Panel and pathogen culture were compared to those obtained from first-generation sequencing.

### Statistical analysis

Continuous variables were summarized as mean ± standard deviation or median (interquartile range). Categorical variables were displayed as frequencies and percentages using the chi-square test or Fisher’s exact test. Evaluation indexes of test results include positive percent agreement (sensitivity), negative percent agreement (specificity), total percent agreement (accuracy), and 95% confidence interval; consistency evaluation of examination reagents and comparison methods: kappa consistency test. All analyses were performed using SPSS software version 26.0 (IBM Corporation, USA). All statistical tests were two-sided and generally done at the 0.05 level, and when *P* < 0.05, the difference between the two tests was considered statistically significant.

## RESULTS

### Demographic and characteristics of the enrolled patients

This study was conducted at Children’s Hospital of Shanghai (Center 01), West China Second Hospital, Sichuan University (Center 02), and Children’s Hospital affiliated with Capital Institute of Pediatrics (Center 03). A total of 1,540 subjects were screened; 1,404 valid cases were enrolled and 136 cases were excluded. Among the excluded cases, 91 of them did not agree to sign the informed consent, 16 of them were repeatedly enrolled, 10 of them did not meet the final clinical diagnosis, 4 of them whose samples were non-standard, and 10 of them failed the test.

A total of 1,136 prospective samples were screened; 34 samples were excluded and 1102 samples were collected finally. Totally 404 retrospective frozen samples were screened, 102 were excluded, and 302 retrospective frozen samples were collected. A total of 1,404 valid cases were included, including 607 females (43.2%) and 797 males (56.8%). Of the prospective samples, 914 (82.9%) were from outpatients and 188 from inpatients (17.1%). Demographics and positivity rates for all prospective and retrospective samples and by age group are shown in Table 1. The median age of the subjects was 3 years old (interquartile range, 1–5 years old), the youngest age was less than 1-month old, and the maximum age was 15 years old.

**TABLE 1:** Demographics and positivity rates for all prospective and retrospective samples and by age group

Parameter	All (*N* = 1,404)		Prospective sample (*N* = 1,102)		Retrospective sample (*N* = 302)	
	No.	%	No.	%	No.	%
Demographics and location						
Male	797	56.8	632	57.4	165	54.6
Female	607	43.2	470	42.6	137	45.4
Outpatients	914	65.1	914	82.9	–	–
Hospitalized	188	13.4	188	17.1	–	–
Overall positivity and codetections						
Negative samples	177	12.6	152	13.8	25	8.3
Positive samples	1227	87.4	950	86.2	277	91.7
Single detections	571	40.7	394	35.8	177	58.6
Codetections	656	46.7	556	50.5	100	33.1
Positivity by age grouping						
<6 mo	120	8.5	92	8.3	28	9.3
7–12 mo	71	5.1	56	5.1	15	5.0
1–3 yr	503	35.8	410	37.2	93	30.8
4–6 yr	390	27.8	305	27.7	85	28.1
7–15 yr	143	10.2	87	7.9	56	18.5

^*a*^ –, N/A.

### Comparison of the amount of pathogen detected between Acaruiter Respiratory Panel and Health Respiratory Panel

**Fig 3:**
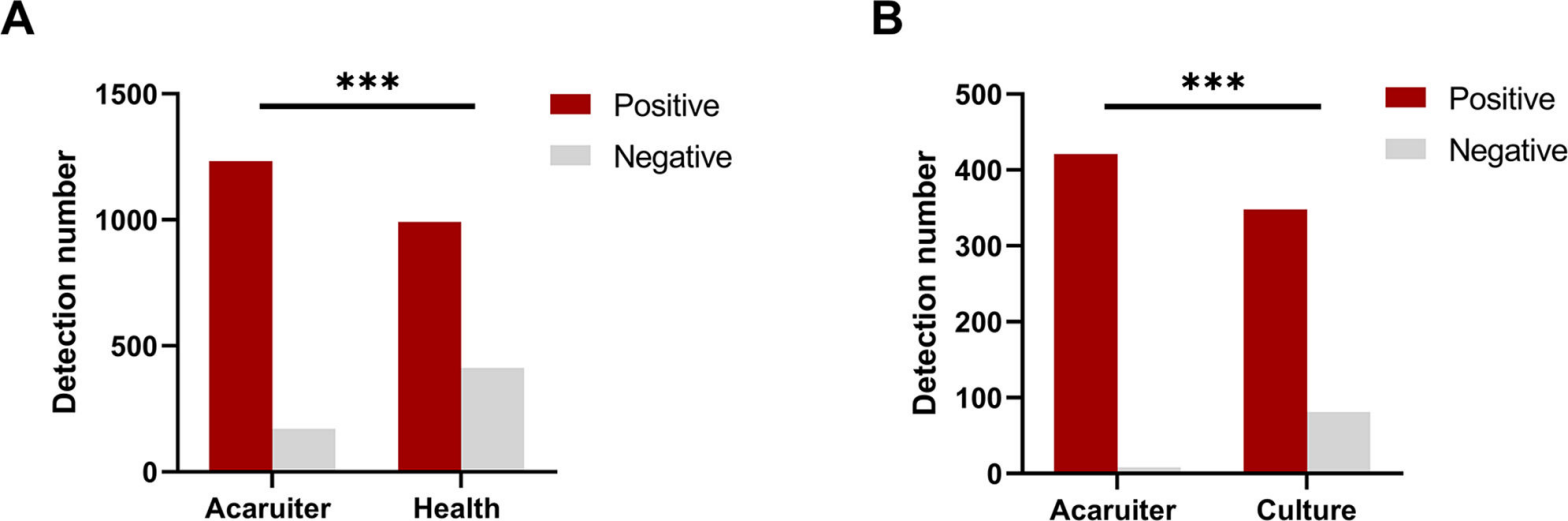
Comparison of the amount of pathogen detected between Acaruiter Respiratory Panel and compared method: Health Respiratory Panel (A) and pathogen culture (B). ***, *P* < 0.001.

One thousand four hundred four valid samples were obtained in this study. Among them, 1,233 positive samples and 171 negative samples were detected by Acaruiter Respiratory Panel, and 992 positive samples and 412 negative samples were detected by Health Respiratory Panel. The pathogen-positive rate of Acaruiter Respiratory Panel was 87.82%, and the pathogen-positive rate of Health Respiratory Panel was 70.66%. The pathogen detection rate of Acaruiter Respiratory Panel was significantly higher than that of Health Respiratory Panel (*χ* ^2^ = 125.73, *P* < 0.001) (Fig. 3A).

### Comparison of the amount of pathogen detected between Acaruiter Respiratory Panel and pathogen culture

Totally, 429 samples were subject to Acaruiter Respiratory Panel and pathogen culture and determined by first-generation sequencing. Among them, 421 positive samples and 8 negative samples were detected by Acaruiter Respiratory Panel, and 348 positive samples and 81 negative samples were detected by pathogen culture. The pathogen-positive rate of Acaruiter Respiratory Panel was 98.16%, and the pathogen-positive rate of pathogen culture was 81.11%. The pathogen detection rate of Acaruiter Respiratory Panel was significantly higher than that of the pathogen culture (*χ* ^2^ = 66.806, *P* < 0.001) (Fig. 3B).

### Spectrum of pathogens

**Fig 4:**
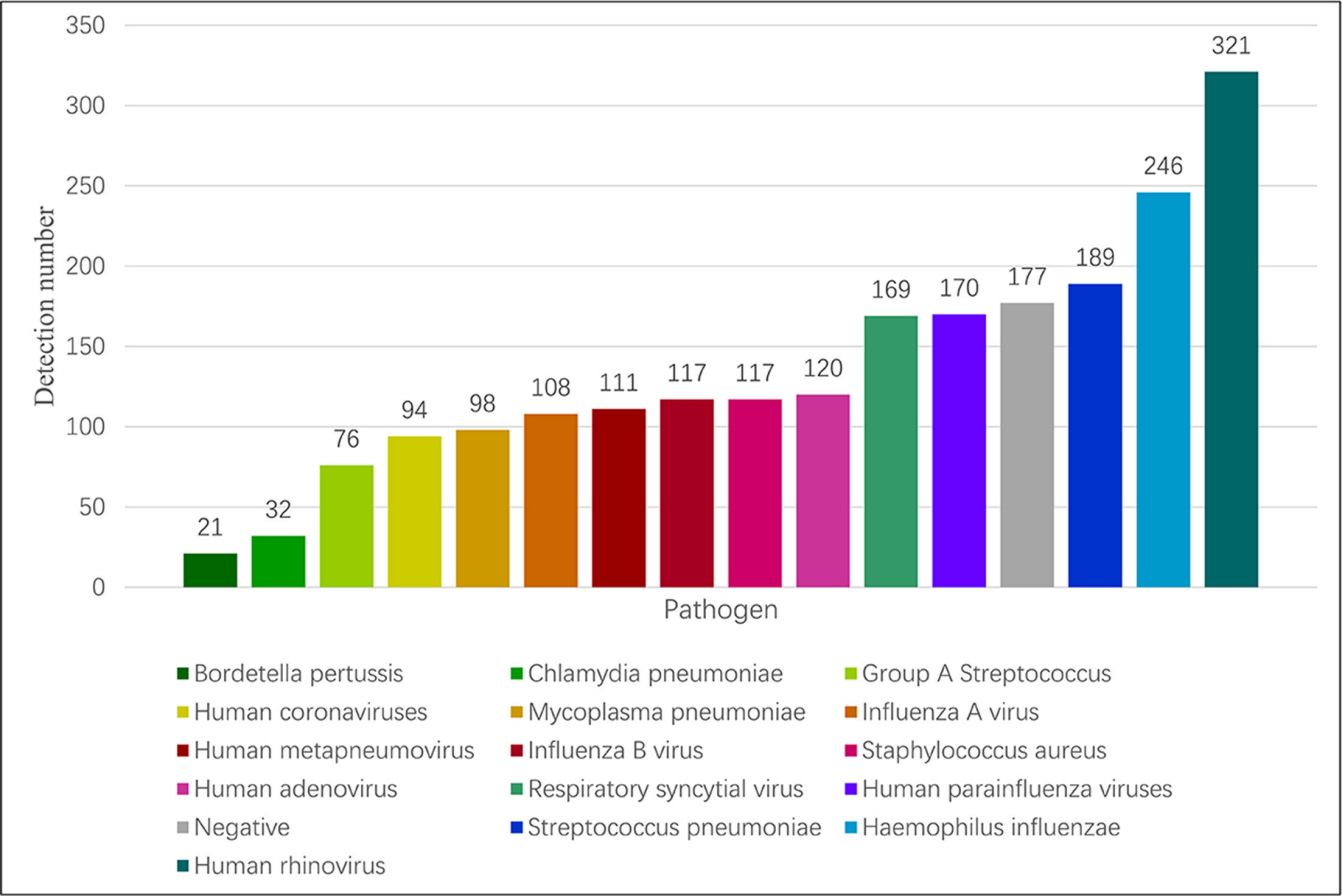
Detection number of each pathogen in all specimens.

**Fig 5:**
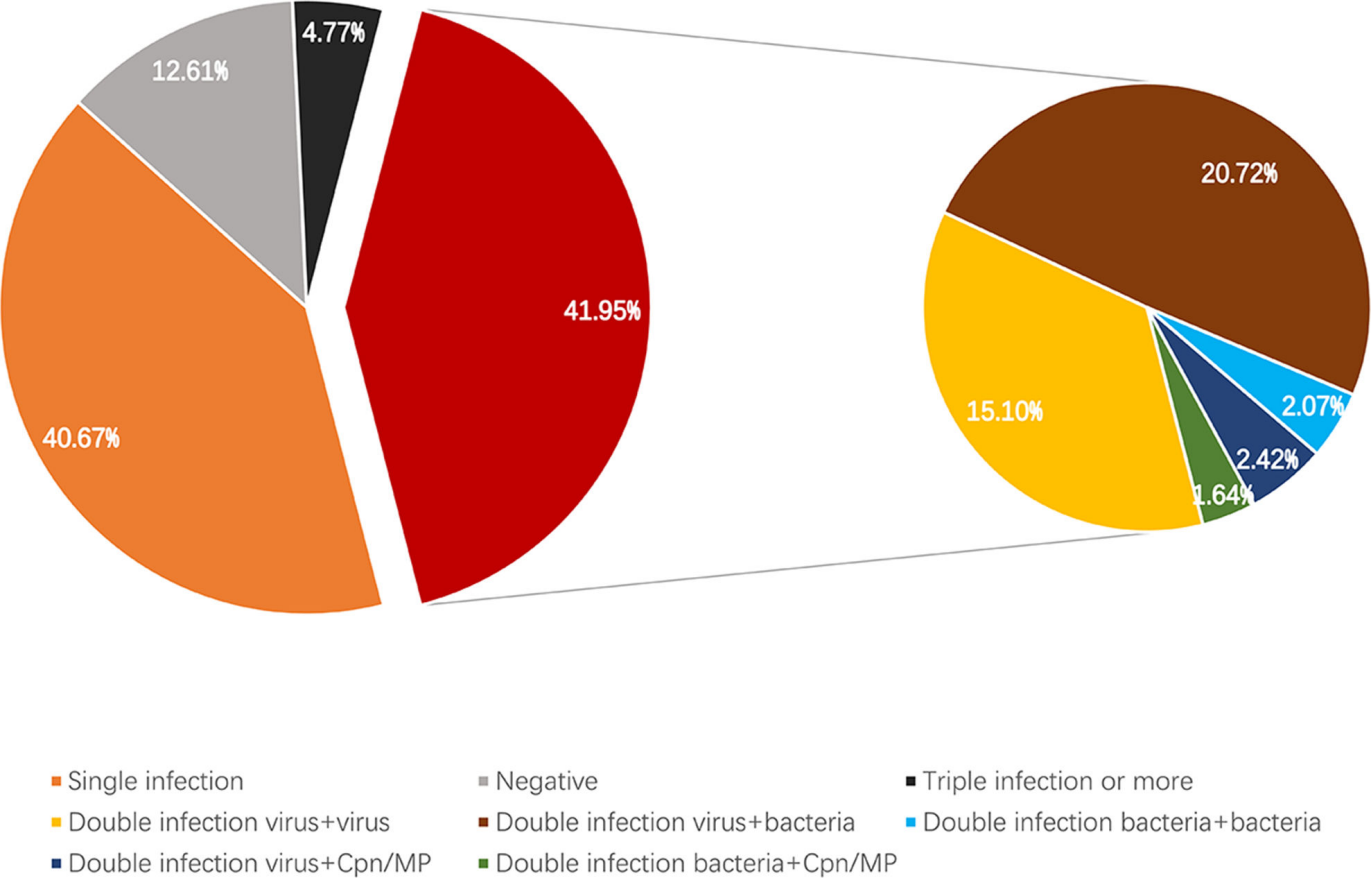
Proportion of infections with different combinations of pathogens.

The test results of 1,404 pharyngeal swab samples were analyzed. There were 177 negative samples and 1,296 positive samples, accounting for 12.6% and 87.4%, respectively. Among the positive samples, there were 571 cases of single infection and 656 cases of mixed infection (629 cases of double infection and 79 cases of triple or more mixed infection), accounting for 40.7% and 46.7% of the positive samples, respectively. The spectrum of each pathogen is shown in Fig. 4. Mixed infections are more common than single infections, as shown in Fig. 5, in which co-infections of bacteria and viruses account for 20.72% (291/1,404).

### Analysis of the coincidence rate of the detection results of pathogens between Acaruiter Respiratory Panel and comparator testing

Compared with comparator testing, the detection results of each pathogen were statistically analyzed. The positive and negative percent agreements of each pathogen were calculated respectively, and the kappa consistency test was carried out. The specific statistical analysis results are shown in Table 2. In the prospective samples and retrospective frozen samples, the positive percent agreement of Acaruiter Respiratory Panel was 97.04%–100%, the negative percent agreement was 99.31%–100%, the total percent agreement was 99.36%–100%, and the kappa value was 0.982–1.000. We further analyzed the prospective samples and the retrospective frozen samples, respectively. In the prospective samples, as shown in Table 3, the positive percent agreement of Acaruiter Respiratory Panel was 96.99%–100%, the negative percent agreement was 99.23%–100%, the total percent agreement was 99.36%–100%, and the kappa value is 0.972–1.000. In the retrospective frozen samples, as shown in Table 4, the positive percent agreement of Acaruiter Respiratory Panel was 91.30%–100%, the negative percent agreement was 99.60%–100%, the total percent agreement was 99.34%–100%, and the kappa value was 0.951–1.000.

**TABLE 2:** Performance summary of the Acaruiter Respiratory Panel for all specimens

Pathogen	TP/(TP + FN)^*a*^	Sensitivity (%)	95% CI^*a*^ (%)	TN/(TN + FP)^*a*^	Specificity (%)	95% CI (%)	Accuracy (%)	Kappa
Viruses								
Influenza A virus	107/108	99.07	94.20–99.95	1,295/1,296	99.92	99.50–100.00	99.86	0.990
Influenza B virus	114/117	97.44	92.13–99.34	1,287/1,287	100.00	99.63–100.00	99.79	0.986
Human adenovirus	118/120	98.33	93.50–99.71	1,284/1,284	100.00	99.63–100.00	99.86	0.991
Human rhinovirus	312/321	97.20	94.56–98.63	1,083/1,083	100.00	99.56–100.00	99.36	0.982
Human parainfluenza viruses	166/170	97.65	93.70–99.25	1,234/1,234	100.00	99.61–100.00	99.72	0.986
Human coronaviruses	92/94	97.87	91.79–99.63	1,310/1,310	100.00	99.64–100.00	99.86	0.988
Respiratory syncytial virus	164/169	97.04	92.86–98.90	1,235/1,235	100.00	99.61–100.00	99.64	0.983
Human metapneumovirus	108/111	97.30	91.72–99.30	1,293/1,293	100.00	99.63–100.00	99.79	0.985
Atypical pathogen								
*Mycoplasma pneumoniae*	98/98	100.00	95.30–100.00	1,306/1,306	100.00	99.63–100.00	100.00	1.000
*Chlamydia pneumoniae*	32/32	100.00	86.66–100.00	1,372/1,372	100.00	99.65–100.00	100.00	1.000
Bacteria								
*Haemophilus influenzae*	244/246	99.19	96.78–99.86	1,150/1,158	99.31	98.59–99.68	99.29	0.976
*Group A Streptococcus*	75/76	98.68	91.88–99.93	1,327/1,328	99.92	99.50–100.00	99.86	0.986
*Bordetella pertussis*	21/21	100.00	80.76–100.00	1,383/1,383	100.00	99.65–100.00	100.00	1.000
*Staphylococcus aureus*	116/117	99.15	94.64–99.96	1,285/1,287	99.96	99.37–99.97	99.86	0.986
*Streptococcus pneumoniae*	188/189	99.47	96.63–99.97	1,210/1,215	99.83	98.99–99.85	99.57	0.982

^*a*^ TP, true positive; FN, false negative; TN, true negative; FP, false negative; and CI, confidence interval.

**TABLE 3:** Performance summary of the Acaruiter Respiratory Panel for prospective specimens

Pathogen	TP/(TP + FN)^*a*^	Sensitivity (%)	95% CI^*a*^ (%)	TN/(TN + FP)^*a*^	Specificity (%)	95% CI (%)	Accuracy (%)	Kappa
Viruses								
Influenza A virus	66/66	100.00	93.15–100.00	1,035/1,036	99.90	99.37–99.99	99.91	0.992
Influenza B virus	44/45	97.78	86.77–99.88	1,057/1,057	100.00	99.55–100.00	99.91	0.988
Human adenovirus	77/78	98.72	92.09–99.03	1,024/1,024	100.00	99.53–100.00	99.91	0.993
Human rhinovirus	291/298	98.86	95.01–98.97	804/804	100.00	99.41–100.00	99.36	0.984
Human parainfluenza viruses	159/163	97.55	93.44–99.21	939/939	100.00	99.49–100.00	99.64	0.985
Human coronaviruses	69/71	97.18	89.28–99.51	1,031/1,031	100.00	99.54–100.00	99.82	0.985
Respiratory syncytial virus	161/166	96.99	92.74–98.89	936/936	100.00	99.49–100.00	99.55	0.982
Human metapneumovirus	86/88	97.73	91.26–99.61	1,014/1,014	100.00	99.53–100.00	99.82	0.988
Atypical pathogen								
*Mycoplasma pneumoniae*	43/43	100.00	89.79–100.00	1,059/1,059	100.00	99.55–100.00	100.00	1.000
*Chlamydia pneumoniae*	18/18	100.00	78.12–100.00	1,084/1,084	100.00	99.56–100.00	100.00	1.000
Bacteria								
*Haemophilus influenzae*	192/194	98.97	95.93–99.82	901/908	99.23	98.35–99.66	99.18	0.972
*Group A Streptococcus*	52/53	98.11	88.62–99.90	1,049/1,049	99.92	99.55–100.00	99.91	0.990
*Bordetella pertussis*	20/20	100.00	79.95–100.00	1,082/1,082	100.00	99.56–100.00	100.00	1.000
*Staphylococcus aureus*	106/107	99.07	94.16–99.95	993/995	99.80	99.19–99.97	99.73	0.985
*Streptococcus pneumoniae*	162/163	99.39	96.12–99.97	934/939	99.47	98.69–99.81	99.46	0.979

^*a*^ TP, true positive; FN, false negative; TN, true negative; FP, false negative; and CI, confidence interval.

**TABLE 4:** Performance summary of the Acaruiter Respiratory Panel for retrospective specimens

Pathogen	TP/(TP + FN)^*a*^	Sensitivity (%)	95% CI^*a*^ (%)	TN/(TN + FP)^*a*^	Specificity (%)	95% CI (%)	Accuracy (%)	Kappa
Viruses								
Influenza A virus	41/42	97.62	85.91–99.88	260/260	100.00	98.19–100.00	99.67	0.986
Influenza B virus	70/72	97.22	89.42–99.52	230/230	100.00	97.95–100.00	99.34	0.982
Human adenovirus	41/42	97.62	85.91–99.88	260/260	100.00	98.19–100.00	99.67	0.986
Human rhinovirus	21/23	91.30	70.49–98.48	279/279	100.00	98.31–100.00	99.34	0.951
Human parainfluenza viruses	7/7	100.00	56.09–100.00	295/295	100.00	98.40–100.00	99.34	1.000
Human coronaviruses	23/23	97.18	82.19–100.00	279/279	100.00	98.31–100.00	100.00	1.000
Respiratory syncytial virus	3/3	96.99	31.00–100.00	299/299	100.00	98.42–100.00	100.00	1.000
Human metapneumovirus	22/23	95.65	76.03–99.77	279/279	100.00	98.31–100.00	99.67	0.976
Atypical pathogen								
*Mycoplasma pneumoniae*	55/55	100.00	91.87–100.00	247/247	100.00	98.09–100.00	100.00	1.000
*Chlamydia pneumoniae*	14/14	100.00	73.24–100.00	288/288	100.00	98.36–100.00	100.00	1.000
Bacteria								
*Haemophilus influenzae*	52/52	100.00	91.43–100.00	249/250	99.60	97.44–99.98	99.67	0.988
*Group A Streptococcus*	23/23	100.00	82.19–100.00	280/281	99.64	97.71–99.98	99.67	0.977
*Bordetella pertussis*	1/1	100.00	5.46–100.00	301/301	100.00	98.43–100.00	100.00	1.000
*Staphylococcus aureus*	10/10	100.00	65.55–100.00	292/292	100.00	98.38–100.00	100.00	1.000
*Streptococcus pneumoniae*	26/26	100.00	83.98–100.00	276/276	100.00	98.29–100.00	100.00	1.000

^*a*^ TP, true positive; FN, false negative; TN, true negative; FP, false negative; and CI, confidence interval.

### Discrepant investigation

In this clinical trial, Health Respiratory Panel was used as the main object of comparator testing. The pathogens that can be detected by the two assays include influenza A virus, influenza B virus, human adenovirus, human rhinovirus, human parainfluenza virus, human coronavirus, respiratory syncytial virus, human metapneumovirus, *Mycoplasma pneumoniae*, and *Chlamydia pneumoniae*. The consistent detection results obtained from both methods align with the results obtained from first-generation sequencing as well. Inconsistent detection of various pathogens is shown in Table 5, with a total of 59 inconsistencies in the detection of specific pathogens. After the verification of first-generation sequencing, there were 2 false positive results and 24 false negative results of Acaruiter Respiratory Panel. There were 3 false positive results and 30 false negative results of Health Respiratory Panel.

**TABLE 5:** Performance summary and characteristics of the Acaruiter RP versus the Health RP and discordant results confirmed by Sanger Sequencing

Pathogen	Acaruiter RP compared to Health RP^*a*^				Confirmed by Sanger Sequencing			
	+/+	+/−^*a*^	−/+	−/−	+/−/+	+/−/−^*a*^	−/+/+	−/+/−
Viruses								
Influenza A virus	105	3	1	1,295	2	1	1	0
Influenza B virus	100	14	3	1,287	14	0	3	0
Human adenovirus	116	2	2	1,284	2	0	2	0
Human rhinovirus	311	1	10	1,082	1	0	8	2
Human parainfluenza viruses	163	3	5	1,233	2	1	4	1
Human coronaviruses	84	2	2	1,310	2	0	2	0
Respiratory syncytial virus	163	1	3	1,273	1	0	3	0
Human metapneumovirus	103	5	1	1,295	5	0	1	0
Atypical pathogen								
*Mycoplasma pneumoniae*	98	0	0	1,306	0	0	0	0
*Chlamydia pneumoniae*	31	1	0	1,372	1	0	0	0
Total	1,274	32	27	12,737	30	2	24	3

^*a*^ RP, Respiratory Panel; +, positive; and −, negative. +/+/+, the first symbol means the result of Acaruiter Respiratory Panel, the second symbol means the result of Health Respiratory Panel, and the third symbol means the result of first-generation sequencing.

Furthermore, inconsistent detection of various pathogens by Acaruiter Respiratory Panel and pathogen culture is shown in Table 6, with a total of 120 inconsistencies in the detection of specific pathogens. After the verification of first-generation sequencing, there were 3 false positive results and 2 false negative results of Acaruiter Respiratory Panel. However, there were 7 false positive results and 108 false negative results of pathogen culture.

**TABLE 6:** Performance summary and characteristics of the Acaruiter RP versus pathogen culture and discordant results confirmed by Sanger Sequencing

Pathogen	Acaruiter RP compared to Health RP^*a*^				Confirmed by Sanger Sequencing			
	+/−^*a*^	+/−^*a*^	−/+	−/−	+/−/+^*a*^	+/−/−	−/+/+	−/+/−
Viruses								
Influenza A virus	40	2	6	381	2	0	1	5
Influenza B virus	57	0	3	369	0	0	1	2
Human adenovirus	0	2	0	427	2	0	0	0
Human rhinovirus	0	6	0	423	6	0	0	0
Human parainfluenza viruses	37	9	0	383	9	0	0	0
Human coronaviruses	0	1	0	428	1	0	0	0
Respiratory syncytial virus	3	16	0	410	16	0	0	0
Human metapneumovirus	0	7	0	422	7	0	0	0
Atypical pathogen								
*Mycoplasma pneumoniae*	0	1	0	428	1	0	0	0
*Chlamydia pneumoniae*	0	0	0	429	0	0	0	0
Bacteria								
*Haemophilus influenzae*	59	21	0	349	20	1	0	0
*Group A Streptococcus*	20	8	0	401	8	0	0	0
*Bordetella pertussis*	0	0	0	429	0	0	0	0
*Staphylococcus aureus*	43	16	0	370	16	0	0	0
*Streptococcus pneumoniae*	35	22	0	372	20	2	0	0
Total	294	111	9	6021	108	3	2	7

^*a*^ RP, Respiratory Panel; +, positive; and −, negative. +/+/+, the first symbol means the result of Acaruiter Respiratory Panel, the second symbol means the result of Health Respiratory Panel, and the third symbol means the result of first-generation sequencing.

## DISCUSSION

Respiratory tract infections in children are often associated with respiratory pathogens, including measles virus, influenza virus, varicella zoster virus, coronavirus, respiratory syncytial virus, adenovirus, *Bordetella pertussis*, diphtheria, *Haemophilus influenzae*, Mycobacterium tuberculosis, Neisseria meningitidis, *Streptococcus pneumoniae*, *Mycoplasma pneumoniae*, and *Chlamydia pneumoniae* (10, 12 – 14). A variety of methods can be applied in clinical pathogen detection, and molecular detection methods are the most widely used (15). From a public health point of view, the multiplex assay to detect respiratory tract infections has proved to be a useful tool for seasonal and sporadic surveillance and preparation of outbreaks (16), supporting the effective management of medical resources (17).

Previous multiplex assays for the detection of pathogens of respiratory tract infections mainly focused on viruses (18 – 20), while in our clinical trials, the proportion of bacterial infections was 43.0%. Acaruiter Respiratory Panel detected more pathogens than Health Respiratory Panel. This may be attributed to Acaruiter’s wider pathogen coverage. Some patients infected by *Haemophilus influenzae*, *Group A Streptococcus*, *Bordetella pertussis*, *Staphylococcus aureus*, and *Streptococcus pneumoniae* could not be identified in the primary screening by Health Respiratory Panel. *Haemophilus influenzae* was the most frequently detected bacterium with an infection rate of 17.5% (246/1,404). In addition, the infection rates of *Streptococcus pneumoniae* and *Staphylococcus aureus* were 13.5% (189/1,404) and 8.3% (117/1,404), respectively. Co-infections of bacteria and viruses account for 20.72%. Acaruiter Respiratory Panel covers five kinds of common bacteria, which is more comprehensive in the diagnosis of respiratory tract infection pathogens and more advantageous in the detection of multiple infections. In addition, although some newly developed assays also pay attention to the infection of bacteria, the bacterial coverage is still limited (21 – 23).

In our study, the most common combination of mixed infections was virus and bacterium in children. A range of respiratory viruses had been suggested to interfere with the growth of the other viruses through resource competition, the immune response, or interference through viral proteins (24, 25). This mechanism can explain the current results showing higher viral–bacterial yet lower viral–viral co-infection rate than expected if a random interactive process was assumed. The current findings revealed only a statistical relationship between pathogens that perhaps suggest mechanisms or pathways for future research. Dynamic surveillance of bacterial and viral epidemiology of respiratory tract infections still needs to be implemented. A better understanding of the complex interactions among viruses, bacteria, and viral–bacterial could help to understand respiratory pathogen epidemiology and planning public health strategies for infection control. Hence, a multiplex PCR assay that covers diversified respiratory viruses and bacteria caters to the needs of detection.

So far, among the kits that have been listed in China, Health Respiratory Panel has the widest coverage of pathogens. Health Respiratory Panel is based on the detection system of multiple PCR and capillary electrophoresis depending on PCR laboratory conditions and manual operation. It can simultaneously detect a total of 13 common respiratory pathogens in one reaction, which has the characteristics of high throughput and low cost, but it does not include the detection of common respiratory tract infection bacteria. Considering the detection results of three clinical centers, it was found that viruses, bacteria, and atypical pathogens were covered, and most of them were mixed infections. Simple detection cannot fully reflect the pathogen of patients, and it is impossible to comprehensively evaluate the infection of patients. The pathogen coverage of the Acaruiter Respiratory Panel is wider, which is more in line with the diagnostic needs of respiratory tract infection patients in the emergency department, outpatient department, inpatient department, and fever clinic.

There was no statistically significant difference in the sensitivity and specificity between Acaruiter Respiratory Panel and Health Respiratory Panel, but sensitivity of Acaruiter Respiratory Panel is better statistically compared to the pathogen culture. Acaruiter Respiratory Panel demonstrated broader pathogen coverage compared to Health Respiratory Panel and exhibited higher sensitivity than pathogen culture methods. This regent may be a promising assay for comprehensive testing for respiratory pathogens from nasopharyngeal swab specimens in Chinese children.

This study has several limitations. First of all, since this study is not designed as an epidemiological study, the clinical samples included are not collected continuously throughout the year, and the detected co-infection rate and the frequency of different pathogens in the co-infection samples may not reflect the actual co-infection rate of the patient population at the time of sample collection. Secondly, because several pathogens, such as *Mycoplasma pneumoniae* and *Bordetella pertussis*, are only detected in a small number of clinical samples, the detection performance of Acaruiter Respiratory Panel for these pathogens is still uncertain. Thirdly, Acaruiter Respiratory Panel is a neoteric one, we have only collected preliminary testing data from three locations in mainland China at present. More testing results from additional countries and regions are needed for comparative analysis in future studies.

### Conclusion

In summary, in this first clinical study of the new Acaruiter Respiratory Panel assay performed in three children medical centers providing 1,404 clinical samples for analysis, we observed excellent diagnostic accuracy of the Acaruiter Respiratory Panel assay compared to Health Respiratory Panel and represents a new alternative for multiplex respiratory testing. It is a robust and accurate assay for rapid and comprehensive testing for respiratory pathogens from nasopharyngeal swab specimens. The Acaruiter Respiratory Panel assay could potentially impact positively on antimicrobial stewardship, hospital admittance, and use of side room contact isolation facilities.
